# Switching and emergence of CTL epitopes in HIV-1 infection

**DOI:** 10.1186/1742-4690-11-38

**Published:** 2014-05-21

**Authors:** Chungyong Han, Ai Kawana-Tachikawa, Akihisa Shimizu, Dayong Zhu, Hitomi Nakamura, Eisuke Adachi, Tadashi Kikuchi, Michiko Koga, Tomohiko Koibuchi, George F Gao, Yusuke Sato, Atsushi Yamagata, Eric Martin, Shuya Fukai, Zabrina L Brumme, Aikichi Iwamoto

**Affiliations:** 1 Division of Infectious Diseases, Advanced Clinical Research Center, the Institute of Medical Science, the University of Tokyo, 4-6-1 Shirokanedai, Minato-ku, Tokyo 108-8639, Japan; 2 Department of Infectious Disease Control, the International Research Center for Infectious Diseases, the Institute of Medical Science, the University of Tokyo, Tokyo, Japan; 3 Department of Infectious Diseases and Applied Immunology, Hospital, the Institute of Medical Science, the University of Tokyo, Tokyo, Japan; 4 CAS Key Laboratory of Pathogenic Microbiology and Immunology, Institute of Microbiology, Chinese Academy of Sciences, Beijing, China; 5 Structural Biology Laboratory, Life Science Division, Synchrotron Radiation Research Organization and Institute of Molecular and Cellular Biosciences, the University of Tokyo, Tokyo, Tokyo, Japan; 6 Department of Medical Genome Sciences, Graduate School of Frontier Sciences, The University of Tokyo, Chiba, Japan; 7 Faculty of Health Sciences, Simon Fraser University, Burnaby, BC, Canada; 8 British Columbia Centre for Excellence in HIV/AIDS, Vancouver, BC, Canada; 9 Asian Research Center for Infectious Diseases, the Institute of Medical Science, the University of Tokyo, Tokyo, Japan; 10 Cancer Immunology Branch, Division of Cancer Biology, National Cancer Center, Goyang-si, Gyeongi-do 410-769, Korea

## Abstract

**Background:**

Human Leukocyte Antigen (HLA) class I restricted Cytotoxic T Lymphocytes (CTLs) exert substantial evolutionary pressure on HIV-1, as evidenced by the reproducible selection of HLA-restricted immune escape mutations in the viral genome. An escape mutation from tyrosine to phenylalanine at the 135th amino acid (Y135F) of the HIV-1 *nef* gene is frequently observed in patients with HLA-A*24:02, an HLA Class I allele expressed in ~70% of Japanese persons. The selection of CTL escape mutations could theoretically result in the *de novo* creation of novel epitopes, however, the extent to which such dynamic “CTL epitope switching” occurs in HIV-1 remains incompletely known.

**Results:**

Two overlapping epitopes in HIV-1 *nef*, Nef126-10 and Nef134-10, elicit the most frequent CTL responses restricted by HLA-A*24:02. Thirty-five of 46 (76%) HLA-A*24:02-positive patients harbored the Y135F mutation in their plasma HIV-1 RNA. Nef codon 135 plays a crucial role in both epitopes, as it represents the C-terminal anchor for Nef126-10 and the N-terminal anchor for Nef134-10. While the majority of patients with 135F exhibited CTL responses to Nef126-10, none harboring the “wild-type” (global HIV-1 subtype B consensus) Y135 did so, suggesting that Nef126-10 is not efficiently presented in persons harboring Y135. Consistent with this, peptide binding and limiting dilution experiments confirmed F, but not Y, as a suitable C-terminal anchor for HLA-A*24:02. Moreover, experiments utilizing antigen specific CTL clones to recognize endogenously-expressed peptides with or without Y135F indicated that this mutation disrupted the antigen expression of Nef134-10. Critically, the selection of Y135F also launched the expression of Nef126-10, indicating that the latter epitope is created as a result of escape within the former.

**Conclusions:**

Our data represent the first example of the *de novo* creation of a novel overlapping CTL epitope as a direct result of HLA-driven immune escape in a neighboring epitope. The robust targeting of Nef126-10 following transmission (or *in vivo* selection) of HIV-1 containing Y135F may explain in part the previously reported stable plasma viral loads over time in the Japanese population, despite the high prevalence of both HLA-A*24:02 and Nef-Y135F in circulating HIV-1 sequences.

## Background

Cytotoxic T lymphocytes (CTLs) are key players in the immune control of Human Immunodeficiency Virus 1 (HIV-1), as they recognize virally-derived peptide epitopes presented by HLA class I molecules on the infected cell surface [1,2]. Over the course of infection however, HIV-1 mutations arise within the infected individual, notably in targeted CTL epitopes, that allow the virus to escape immune recognition by CTLs. Importantly, despite the hypermutability of HIV-1, these immune escape mutations often arise in a stereotypical manner [3,4] that is highly predictable based on the specific HLA class I molecules expressed by the host [5-8]. Although selection of HLA-associated mutations in HIV-1 is driven by immune pressure, these amino acid substitutions sometimes result in the induction of a *de novo* immune response in which the mutant epitope is recognized by a TCR associated with a different CTL subset [7,9]. What is less well-characterized is the extent to which selection of immune escape mutations result in the *de novo* creation of novel CTL epitopes nearby, that could subsequently be targeted by CTL *in vivo* (in a manner similar to the continual exposure of novel antibody epitopes in HIV-1 envelope as a consequence of escape from earlier humoral responses [10]). Here, we demonstrate such a dynamic phenomenon of “CTL epitope switching” as a direct result of CTL escape from HLA-A*24:02.

We reported previously that the substitution from tyrosine to phenylalanine (Y135F) at the 135th amino acid of the HIV-1 *nef* gene is frequently observed in patients with HLA-A*24:02, an HLA Class I allele expressed in ~70% of Japanese persons [4,11]. Our observation that Y135F appeared to be an escape mutation was later confirmed [12]. In order to examine the influence of HIV-1 mutations on the strength of various epitope-specific CTL responses, we studied CTL epitopes restricted by HLA-A*24:02 in relatively conserved regions of the HIV-1 genome. Our results indicate that Nef-Y135F, selected to escape recognition of a well-described HLA-A*24:02-restricted CTL epitope in this viral protein, results in the creation of another HLA-A*24:02 epitope immediately upstream. To our knowledge, our findings represent the first evidence of immune escape-driven “epitope switching” in HIV-1 infection.

## Results

### Identification of immunodominant CTL responses restricted by HLA-A*24:02

**Figure 1:**
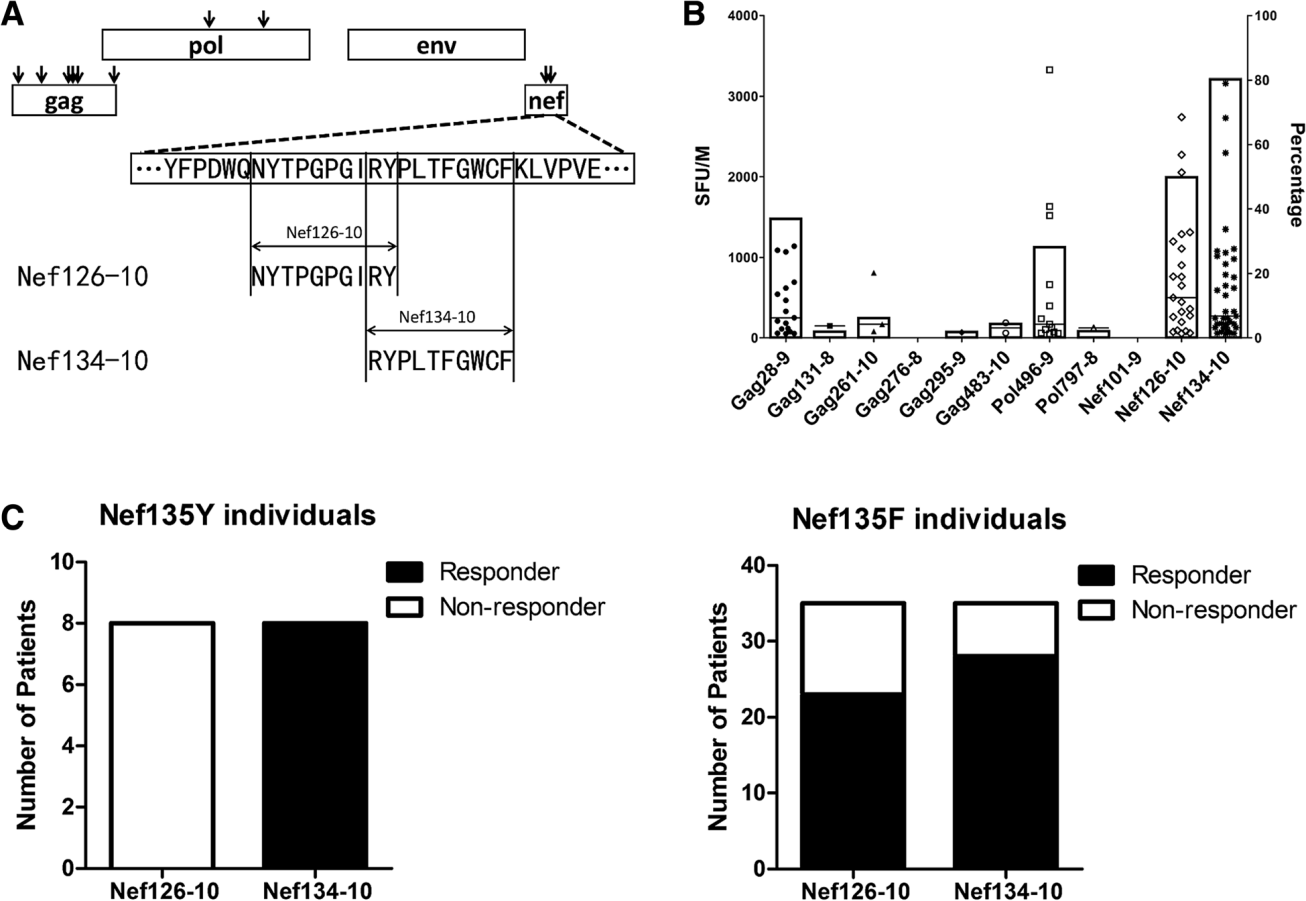
**The association between the two overlapping epitopes and the Y135F mutation. (A)** HIV-1 map of representative proteins and the locations of HLA-A*24:02-restricted epitopes used in this study are shown (arrow). The positional relation and the sequence information around Nef126-10 and Nef134-10 epitopes are also shown. **(B)** Immune responses to 11 epitopes were assessed by IFN-γ ELISpot assay by using expanded PBMCs from 46 HIV-1 patients. Epitope names below the chart indicate peptides used in the assay; [Protein][Location(HXB2 numbering)]-[Amino acid length]. Each point represents the average SFU of duplicate wells after subtraction of background in each individual. Each stick represents response rate in 46 individuals. **(C)** The Nef126-10/Nef134-10-specific response rates among individuals harboring either Nef135Y or Nef135F were assessed. Black bars indicate number of responders to each epitope, and white bars for non-responders.

Forty-six HLA-A*24:02-positive patients with HIV-1 infection were studied. Forty-four were infected through unprotected sexual intercourse and 2 were hemophiliacs. Forty-five were infected with subtype B except one was infected with subtype AG. The median plasma viral load (pVL) was 4.11 (range 2.26 to 5.36) log 10 copies/ml, and the median CD4 cell count was 395 (range 120 to 1,035) cells/μl. To determine which published HLA-A*24:02-restricted CTL epitopes are most frequently recognized among persons expressing this allele, IFN-γ ELISpot assays were performed using expanded PBMCs. Due to limited PBMC numbers, 11 published A*24:02-restricted CTL epitopes in the relatively conserved *gag*, *pol* and *nef* regions [13-15] were selected for investigation. Published optimal epitopes were used for the assay. The response rate against Nef134-10 was highest (80.4%), followed by Nef126-10 (50.0%), Gag28-9 (40.0%) and Pol496-9 (28.3%), while limited (<10%) or no responses were observed in the other epitopes (Figure 1A, B). Of note, Nef126-10 and Nef134-10 overlap each other by 2 amino acids (Figure 1A).

We next analyzed patient plasma HIV RNA amino acid sequences within the Nef126-10–Nef134-10 regions (Table 1). The great majority of patients (35/46 = 76.1%) had a tyrosine (Y) to phenylalanine (F) mutation (Y135F) at Nef codon 135 (Nef135F) while eight patients (8/46 = 17.4%) had the global consensus subtype B residue at this position (Nef135Y). Two were Nef135L and one was Nef135W. These results were consistent with our earlier findings [4]. Intriguingly, none of the eight patients with Nef135Y exhibited a Nef126-10-specific response, while all of them exhibited a Nef134-10-specific response (p < 0.001, Fisher’s exact test) (Figure 1C, left). Of the 35 patients harboring Nef135F, 23 (65.7%) and 28 (80.0%) responded to Nef126-10 and Nef134-10, respectively (Figure 1C, right) (p = 0.2823, Fisher’s exact test).

**Table 1:** Amino acid sequences (Nef126-143) of plasma HIV-1 in 46 patients

**Group**^**a**^	**Amino acid sequence**^**b**^	**Frequency (number)**^**c**^
**Consensus B**	**NYTPGPGIRYPLTFGWCF**	
135Y group		*17.4% (8)*
	..................Y	2.2% (1)
	.............L....	4.3% (2)
	.............L...Y	2.2% (1)
	.......V..........	2.2% (1)
	C......T.....L....	2.2% (1)
	.......T........P.	2.2% (1)
	.......T....C.....	2.2% (1)
135F group		*76.1% (35)*
	.........F........	4.3% (2)
	C........F........	2.2% (1)
	G........F........	2.2% (1)
	.........F..C.....	2.2% (1)
	.......T.F........	50.0% (23)
	C......T.F........	2.2% (1)
	G......T.F........	2.2% (1)
	.......V.F........	8.7% (4)
	.......E.F..C.....	2.2% (1)
others		*6.5% (3)*
	.......V.L........	4.3% (2)
	.......T.W........	2.2% (1)

^a^Patients were partitioned into three groups, Nef135Y (135Y), Nef135F (135F), or others, according to their amino acid information at the Nef135 position.

^b^Middle column shows amino acid sequence of the Nef126-143 region. The same amino acids as the subtype B consensus sequence are indicated by dots. Differences compared to the subtype B consensus sequence are indicated by the corresponding letters.

^c^Right column indicates frequency (and number) of individuals exhibiting the stated sequence. Subtotal frequency (and number) of each group is italicized.

### Dramatic improvement in the HLA-binding affinity of Nef126-10 following mutation of the C-terminal anchor residue

**Figure 2:**
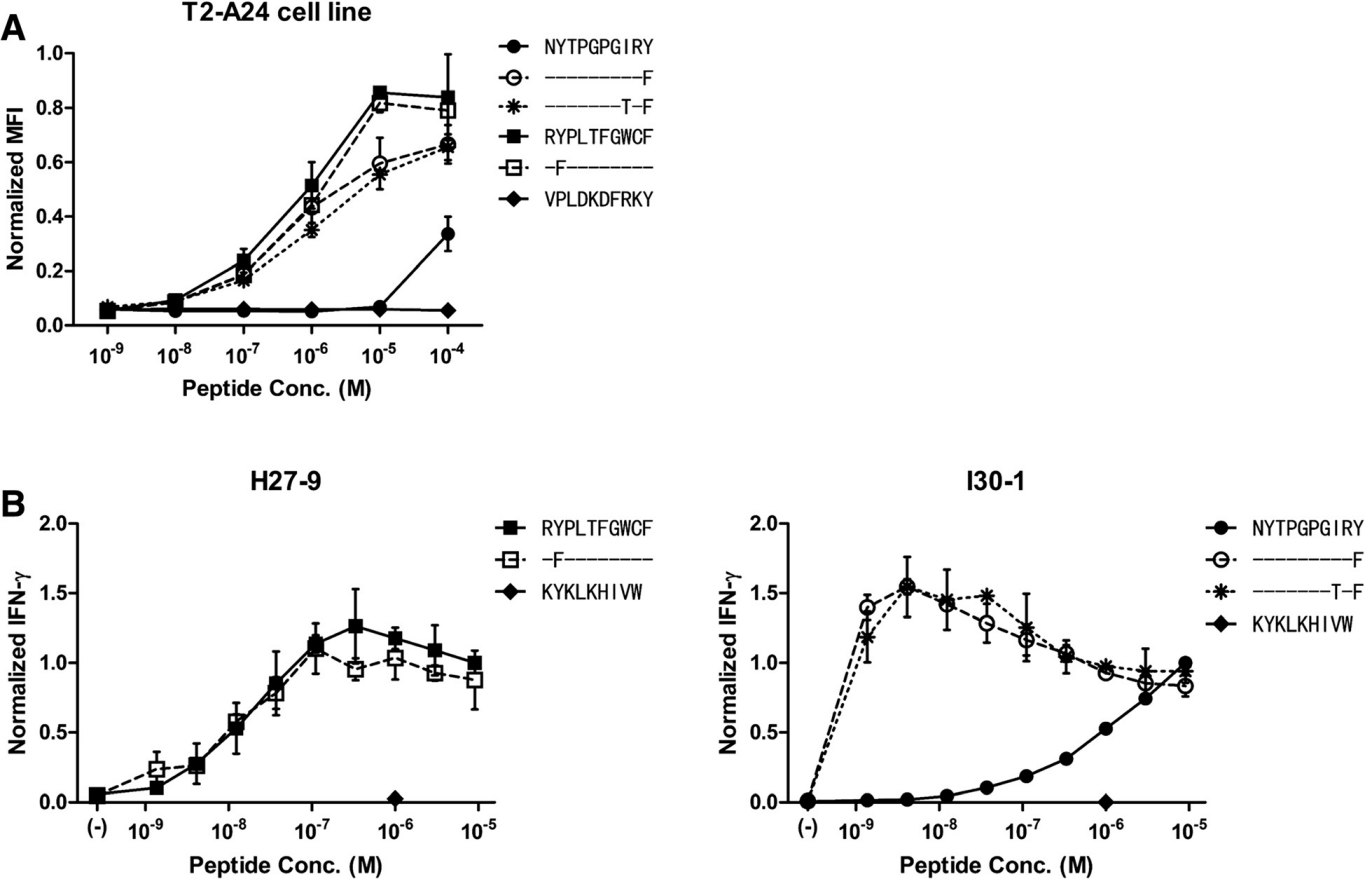
**Epitope characterization. (A)** Binding affinity of each peptide to HLA-A*24:02 was tested by using T2-A24 cell line. All peptides except negative control peptide, VPLDKDFRKY, which might not bind to HLA-A*24:02, show HLA-A*24:02 binding in the peptide concentration of 10^-4^ M. Each point and bar represents the average and the standard deviation of normalized MFI from three independent experiments. **(B)** 293FT-A24DRm-CY0 cells were pulsed with serially diluted peptides and co-cultured with CTL clones specific to Nef134-10 (H27-9) or Nef126-10 (I30-1). Epitope recognition was determined by normalizing quantity of IFN-γ secretion. For normalization, all values were divided by IFN-γ secretion with wild type peptide concentration of 9 μM. Neither clone showed specificity to the negative control, Gag28-9 peptide (KYKLKHIVW) a binder of HLA-A*24:02. Each point and bar show average and standard deviation of three independent experiments.

To clarify the relationship between Y135F and peptide-specific responses, we examined HLA-binding affinity of the wild type and mutant peptides using *in vitro* peptide-HLA binding assays (Figure 2A). In context of the Nef134-10 epitope, the mutant Y135F peptide (representing position 2, the N-terminal anchor of this epitope; Nef134-10(2F)) was almost as effective as the “wild type” Nef134-10 (Nef134-10(wt)) peptide in binding to HLA-A*24:02. In contrast, in context of the Nef126-10 epitope, the mutant Y135F peptide (representing position 10, the C-terminal anchor of this epitope), dramatically improved its binding to HLA-A*24:02. The presence of threonine (T) at the 8th position (Nef126-10(8T10F)), representing Nef mutation I133T, did not significantly affect epitope-HLA binding compared to the wild type isoleucine (I) (Nef126-10(8I10F)). These results are compatible with previous reports identifying Y or F as possible N-terminal anchors for HLA-A*24:02, but only F as a possible C-terminal anchor [16,17].

We then examined the effect of the mutations on epitope recognition using CTL clones established from patients with HIV-1 infection. 293FT-A24DRm-CY0 cells pulsed with different dilutions of peptides were co-cultured with CTL cell clones. The Nef134-10-specific CTL clone H27-9 produced IFN-γ almost equally well in response to Nef134-10(wt) peptides or to Nef134-10(2F) peptides (Figure 2B, left). In contrast, the Nef126-10-specific CTL clone I30-1 produced IFN-γ only at high concentrations of the wild-type Nef126-10(wt) peptide, whereas mutant peptides Nef126-10(8I10F) and Nef126-10(8T10F) induced strong responses at very low peptide concentration (Figure 2B, right). These results were consistent with peptide-HLA binding assays suggesting that the I133T mutation did not have much effect on recognition of the epitope-HLA complex by the Nef126-10-specific CTL clone I30-1. Moreover, the results were consistent with the observation that the presence of wild-type Y at the C-terminus lowers the affinity of the Nef126-10 peptide to HLA-A*24:02 (Figure 2A).

### CTL responses against the endogenously expressed epitopes

**Figure 3:**
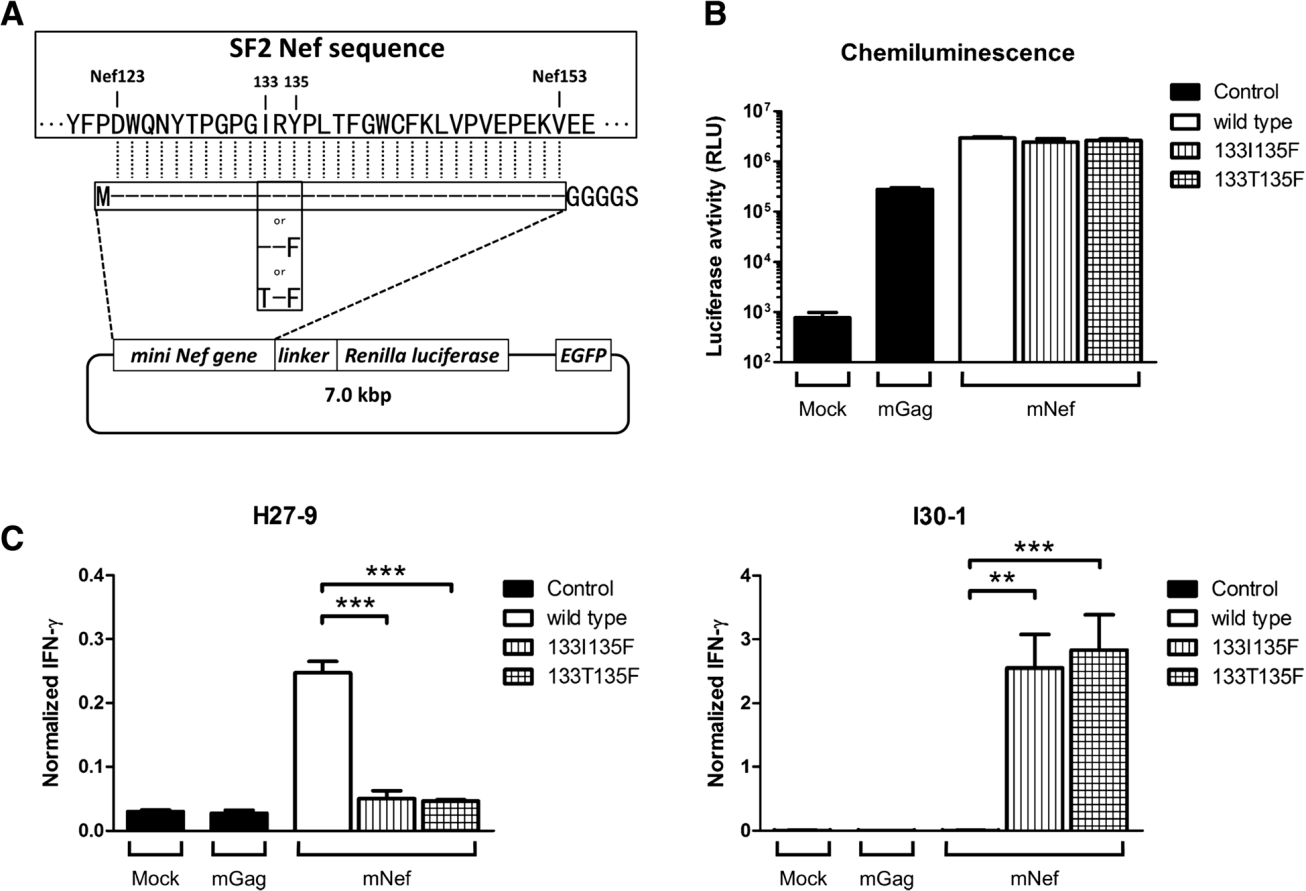
**Recognition of endogenously derived epitope. (A)** Vector construction. The vector expresses EGFP as a transfection marker and a fusion protein that consists of mini-Nef gene coding 31 amino acids of Nef123-Nef153 region and GlyGlyGlyGlySer linker and renilla luciferase. The 133I/135F and 133T/135F mutant vectors as well as wild type vector were constructed. Mini-Nef sequence that corresponds to the actual Nef gene is shown. **(B)** By measuring luciferase activity, quantity of the generated mini-Nef was assessed. No significant differences in mini-Nef generation between wild type, 135F and 133T/135F were observed. Each stick and bar indicates average and standard deviation of three independent experiments. **(C)** The vector transfected cells were incubated with either H27-9 or I30-1, and IFN-γ secretion was quantified by ELISA and normalized to determine the epitope recognition. For normalization, each value was divided by IFN-γ secretion with wild type peptide concentration of 9 μM. Mock as well as mGag, transfected with mGag(wt)-hRluc-EGFP, were measured as negative controls. Each stick and bar indicates average and standard deviation of three independent experiments. The significance was calculated by unpaired Student’s t-test; **, p < 0.01; ***, p < 0.001.

In order to examine whether intracellularly-derived Nef protein could still be targeted by peptide-specific CTLs, we constructed *nef*-minigene expression vectors, pmNef(wt)-hRluc-EGFP, pmNef(135F)-hRluc-EGFP, and pmNef(133T135F)-hRluc-EGFP, for the generation of polypeptides encompassing the Nef126-10 and Nef134-10 epitopes (Figure 3A). The vectors encoded EGFP as a transfection marker, as well as the *Renilla* Luciferase (Rluc) gene hooked to the mini-*nef* gene by a GlyGlyGlyGlySer linker. Rluc activity served as a quantitative reference for the expression of the mini-*nef* polypeptide. Each vector was transfected into 293FT-A24DRm-CY0 cells. Rluc activities indicated that three types of *nef*-minigenes were expressed well and to comparable levels (Figure 3B).

We and others reported previously that Y135F is a processing mutation, as CTL responses could be induced to mutant epitopes via peptide-pulsing, but not via intracellularly-expressed polypeptide [4,12,18]. Consistent with the previous results, Nef134-10-specific responses by CTL clone H27-9 were induced by the wild type minigene, but diminished to minimal levels by the presence of Y135F or I133T/Y135F (Figure 3C, left).

By contrast, Nef126-10-specific responses by CTL clone I30-1 were provoked dramatically by the presence of Nef135F. Specifically, the Nef126-10-specific CTL clone I30-1 showed much higher responses to antigen-presenting cells transfected with the 133I/135F or 133T/135F minigene than Nef134-10-specific CTL clone H27-9. The I30-1 responses to minigenes encoding I versus T at the Nef133 position did not substantially differ (Figure 3C, right). In contrast, I30-1 responses to the wild type minigene were indistinguishable from background. These results suggest that wild-type Nef126-10 peptide was not expressed as an epitope on the surface of the antigen-presenting cells when expressed endogenously, but Nef126-10 containing 135F (regardless of variation at position 133) was efficiently expressed. In turn, these *in vitro* results (Figure 2 and 3) strongly suggest that a novel mechanism, i.e. “epitope switching” was taking place after the selection of the Y135F mutation *in vivo* (Figure 1). Namely, selection of Y135F facilitates escape from CTL responses targeting the first epitope (Nef134-10), but simultaneously results in the creation of another epitope upstream (Nef126-10).

### “Epitope switching” during the clinical course of HIV-1 infection

**Figure 4:**
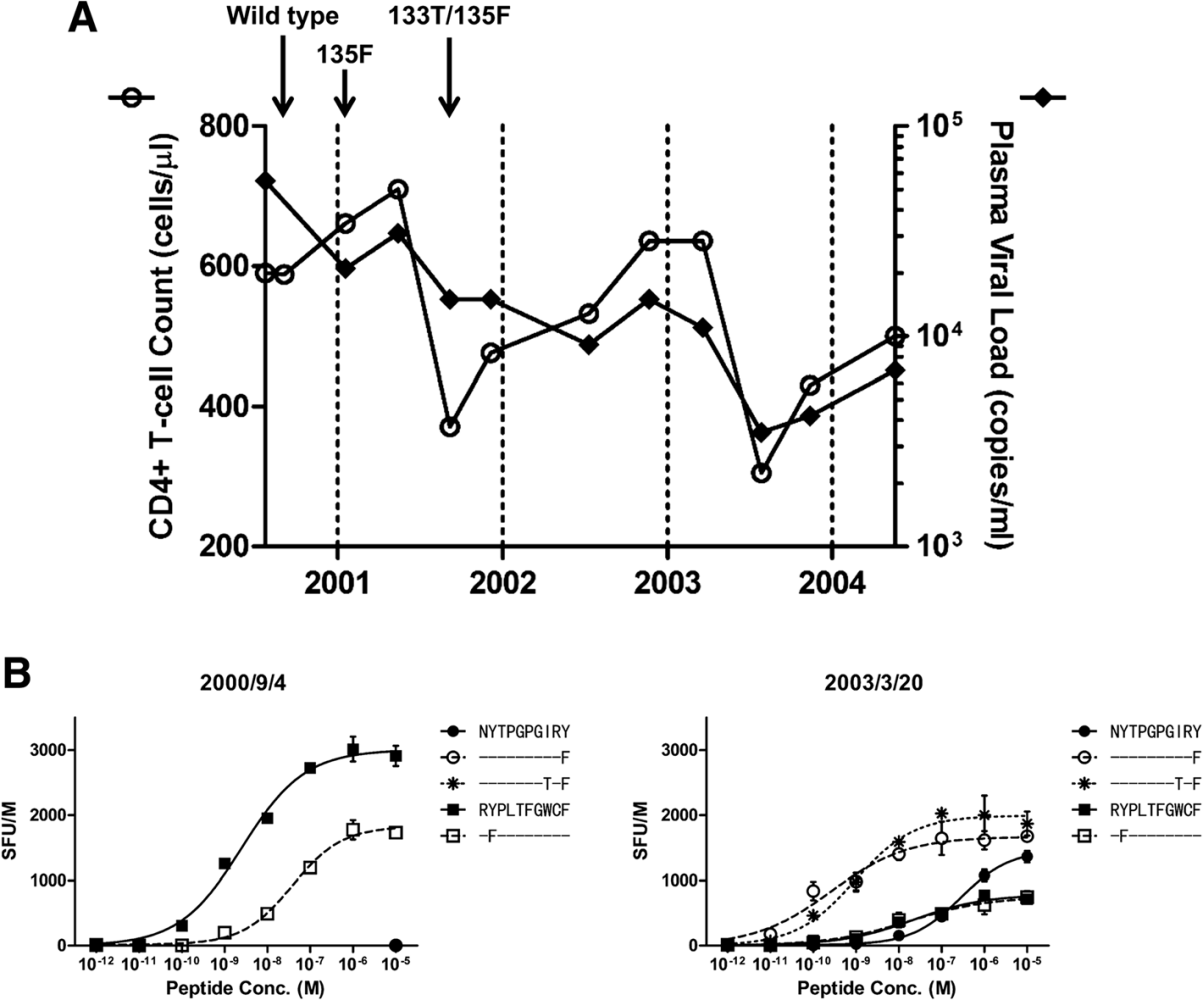
**Longitudinal analysis in one individual. (A)** Time courses of the surrogate markers are shown. CD4+ T cell count (left axis, white circle) and pVL (right axis, black circle) are plotted on the chart. Each arrow indicates the earliest time point when the sequence above the arrow was observed. **(B)** IFN-γ ELISpot assay was performed by using serially diluted peptide set and expanded PBMCs. Two time points were tested for the assay. At the earlier point, 2000/9/4, the patient harbored wild type sequence around the Nef126-10/Nef134-10 region, and at the latter point, 2003/3/20, the sequence displayed 133T/135F. Mutants as well as wild type peptides of Nef126-10 or Nef134-10 epitopes were used in the assay. Each point represents the average SFU of duplicate wells after subtraction of background, and each error bar shows standard deviation.

Among 8/46 patients in the IMSUT cohort who initially harbored the “wild type” (global consensus B) Y135 residue within the Nef134-10 epitope, we identified one patient who subsequently selected 135F, followed by 133T, over a period of 12 months. We performed IFN-γ ELISpot assays on PBMCs expanded from corresponding frozen longitudinal samples (Figure 4A). Before the mutated viruses became the majority, specific responses to Nef134-10(wt) were the most prominent, followed by responses to Nef134-10(2F) (Figure 4B, left). Importantly, no Nef126-10-specific responses were observed at these time points. After plasma viruses were replaced by viruses with 133T/135F, robust responses against Nef126-10(8I10F) and Nef126-10(8T10F) were observed, while responses against Nef126-10(wt) were detected only at high peptide concentrations (Figure 4B, right). These results were consistent with the results *in vitro* using CTL clones (Figure 2B, right), and support the *in vivo* presentation of Nef126-10 only after selection of Y135F. Of interest, responses against Nef134-10 peptides decreased but remained detectable after the selection of 135F and 133T/135F mutations.

### Coupled selection of Nef135F and Nef133T mutants in vivo

**Figure 5:**
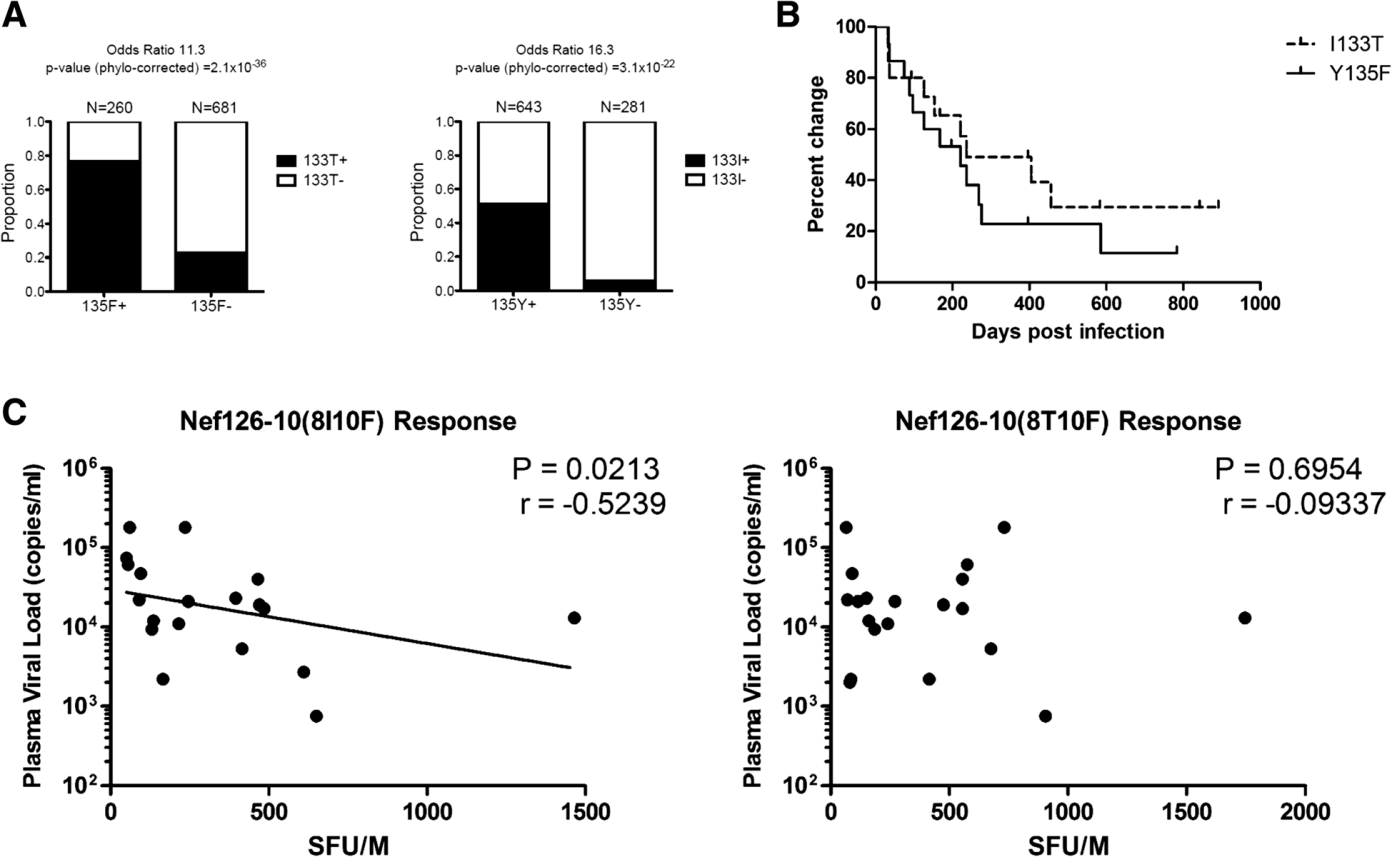
**Relevance of I133T mutation to Y135F and its impact on CTL responses. (A)** The cross-sectional analysis of the co-variation relationship between Nef codons 133 and 135. Nef codons containing gaps or amino acid mixtures were excluded from analysis, leaving 941 and 924 sequences for analysis of codon pairs 135F/133T and 135Y/133I, respectively. **(B)** Kaplan-Meier plot of the emergence of Y135F and I133T mutations in a multicenter longitudinal acute/early infection cohort comprising 16 HLA-A*24:02-expressing persons. **(C)** Within 24 patients who harboring 133I/135F or 133T/135F mutant, correlation between the magnitude of peptide-specific response and pVL was examined. Peptide-specific response was determined by using *ex vivo* IFN-γ ELISpot assay with frozen PBMCs. Each point is plotted according to the average SFU of duplicate wells and the measured pVL at the sampling point. Statistical significance of the correlation between the magnitude and pVL was calculated by Spearman’s rank correlation.

We investigated the correlation between Nef135F and Nef133T *in silico* in two other independent cohorts. In a large cohort of antiretroviral-naïve patients chronically infected with subtype B HIV-1 in British Columbia, Canada (British Columbia HOMER cohort), positive correlations between Nef135F and Nef133T (Odds ratio: 11.3), as well as between Nef135Y and Nef133I (Odds ratio: 16.3) were observed (Figure 5A, all p < 0.0001). Furthermore, in a multicenter longitudinal acute/early infection cohort comprising 16 HLA-A*24:02-expressing persons infected with subtype B HIV-1, selection of Nef135F preceded that of Nef133T by a short duration (Figure 5B). The median times to Y135F and I133T selection were 220 and 236 days, respectively, a difference that was not statistically significant.

The correlation between the magnitude of Nef126-10(8I10F) or Nef126-10(8T10F)-specific response and pVL was assessed in 24 IMSUT cohort participants for whom Nef126-10(8I10F) and Nef126-10(8T10F) responses (measured by IFN-γ ELISpot) and pVL at the corresponding time point, were available (Figure 5C). Interestingly, Nef126-10(8I10F)-specific but not Nef126-10(8T10F)-specific responses were inversely correlated with pVL, suggesting that responses to the former, but not the latter, contribute to *in vivo* immune control.

### Crystal structures of Nef126-10 epitopes presented on HLA-A*24:02

**Figure 6:**
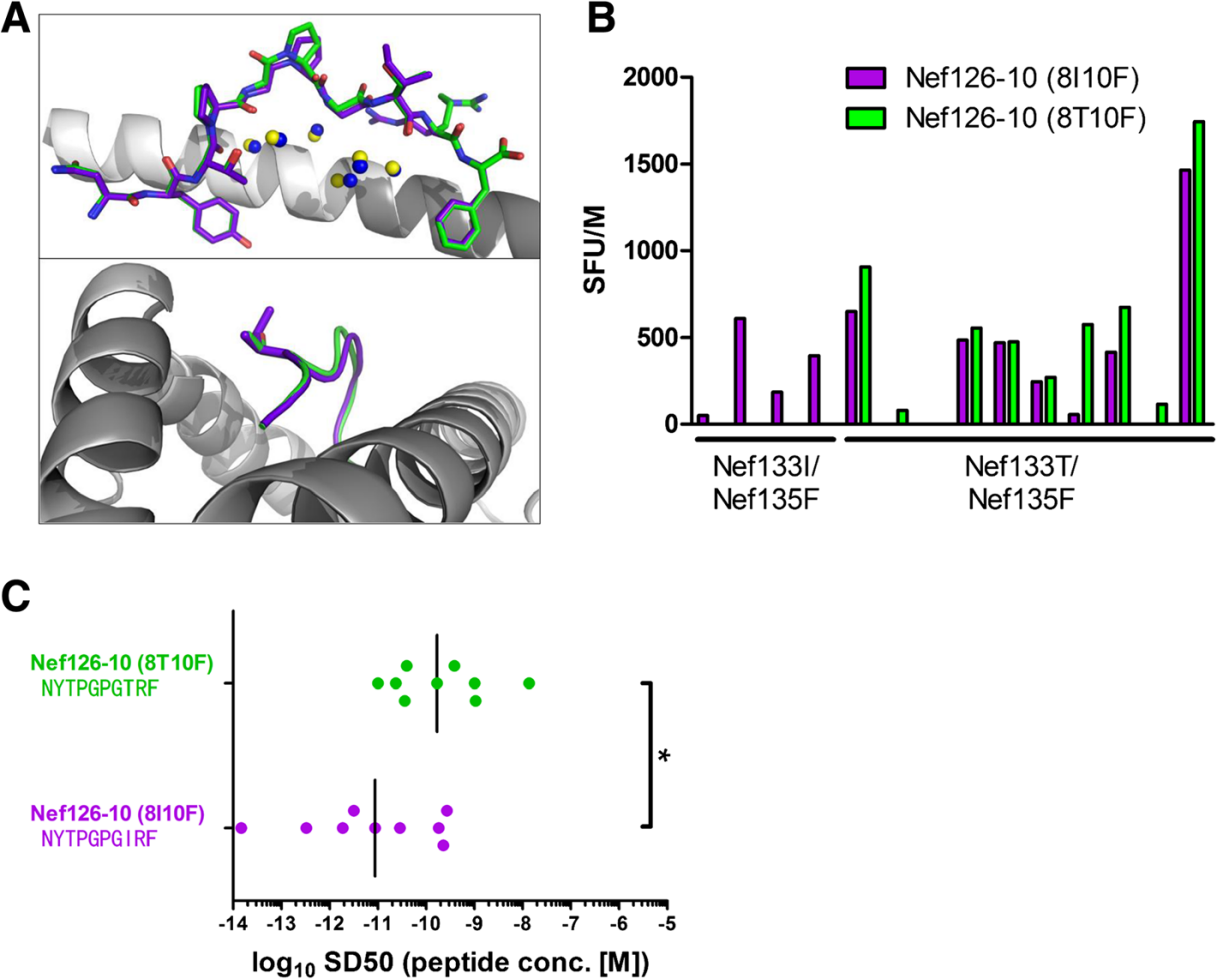
**Impact of I133T mutation on the structures of pHLA and responding T cell subsets. (A)** Top; Side view of structures of the Nef126-10(8I10F) (colored purple) and Nef126-10(8T10F) (colored green) is shown. Superimposition is based on the HLA-A*24:02 peptide binding domain. Water molecules are represented in blue and yellow spheres for the Nef126-10(8I10F) and Nef126-10(8T10F) epitopes, respectively. The number of peptide residues is labeled in black through P1 to P10. The HLA-A*24:02 is represented as a dark grey (Nef126-10(8T10F) and light grey cartoon. **(B)** The presence of the responses to each peptide was examined by *ex vivo* ELISpot assay. A pair of sticks, Nef126-10(8I10F) (purple) and Nef126-10(8T10F) (green), represents the average SFU of duplicate wells to each peptide in one patient. Four individuals on the left side harbored 133I/135F mutant and 10 individuals on the right side harbored 133T/135F mutant. **(C)** Functional avidity curve was drawn for 9 subjects who harbored Nef133T/135F sequence. From the curve, SD50 was calculated and projected in this chart. Nef126-10(8I10F)-specific CTL responses (purple) showed significantly higher functional avidity compared to Nef126-10(8T10F) (green). Each point represents functional avidity for specific peptide in one individual. The significance between two groups was calculated by Mann–Whitney U-test; *, p < 0.05.

In order to examine the impact of these mutations on epitope structure, we solved the crystal structures of HLA-A24/Nef126-10(8I10F) and HLA-A24/Nef126-10(8T10F) at 1.66 Å and 2.0 Å resolution, respectively (Figure 6A top and bottom; Additional file 1: Figure S1A, B). Superposition of the Nef126-10(8I10F) and Nef126-10(8T10F) peptide structures showed almost similar backbone atoms, with root mean square deviation of 0.307 Å, but conformational differences were found at P6 (131P) and P9 (134R) residues. The side chains of P6 and P9 residues in the Nef126-10(8I10F) and Nef126-10(8T10F) epitopes had poor electron densities in spite of structures being at modestly higher resolution (Additional file 1: Figure S1C, D). In addition, the B-factors for the central portions (P5-P7) of each peptide (41.5 Å^2^ for the Nef126-10(8I10F) and 46.2 Å^2^ for Nef126-10(8T10F)) were higher than for overall peptides (24.1 Å^2^ for Nef126-10(8I10F) and 33.2 Å^2^ for Nef126-10(8T10F)). These results indicated a flexibility of the central portion and P9 residue in both peptides, accounting for the structural difference observed.

The side chains of P8-Ile and P8-Thr protruded from, rather than being buried within, the antigen-binding cleft of HLA-A*24:02, suggesting the P8 residue could be involved in the contact with TCR (Figure 6A bottom). Therefore, different TCRs could be favored by the presence of either hydrophobic P8-I or hydrophilic P8-T at the interface of a TCR-HLA-A*24:02/Nef126-10. If this is the case, different TCR repertoires would be selected by Nef126-10(8I10F) or Nef126-10(8T10F), suggesting Nef-I133T as a possible immune escape mutation that alters the *in vivo* repertoire of CTL recognizing this epitope.

### Immune responses against Nef126-10 epitopes

We compared the epitope-specific immune responses between two groups of individuals: those whose plasma viruses were 133I/135F (n = 4) or 133T/135F (n = 10). *Ex vivo* IFN-γ ELISpot assays using PBMCs and Nef126-10(8I10F) or Nef126-10(8T10F) revealed that 0 of 4 patients with Nef126-10(8I10F) viruses had Nef126-10 (8T10F)-specific responses (Figure 6B). Nine out of 10 patients with Nef126-10(8T10F) viruses exhibited specific responses to the circulating epitope, and 7 of 10 patients retained the response specific against Nef126-10(8I10F). These results strongly suggested that the I133T mutation induced a new subset of CD8+ T cells capable of recognizing Nef126-10(8T10F) (p = 0.005, Fisher’s exact test).

Functional avidity has been reported as a correlate of CTL selective pressure [19,20]. As such, we analyzed functional avidities of Nef126-10-specific CTLs. Nine individuals harboring 133T/135F were analyzed by limiting dilution (Figure 6C). Nef126-10(8I10F)-specific CTL responses showed significantly higher avidities compared to those against Nef126-10(8T10F). Taken together with the observation that pVL correlated inversely with the magnitude of Nef126-10(8I10F)-specific, but not Nef126-10(8T10F)-specific, responses (Figure 5C), these results suggest that the new subset of CD8+ T cells elicited following selection of I133T exert less immune pressure on the 133T mutant compared to the “wild-type” I133. The hypothesis that Nef-I133T is an A*24-driven escape mutation is additionally supported by numerous HLA-association studies in HIV subtype B-infected populations including Japan, which consistently demonstrate highly significant associations between A*24 and Nef-I133T [4,8,21,22].

## Discussion

HLA-A*24:02 is highly prevalent among East Asians including Japanese [11]. In an effort to identify immunodominant CTL epitopes presented by HLA-A*24:02, we observed that the two most frequently-recognized epitopes lay in Nef and overlapped each other by two amino acids. Nef codon 135 is critical to both epitopes, as it represents the N-terminal anchor for the downstream epitope Nef134-10, and the C-terminal anchor for the upstream epitope Nef126-10. In the downstream epitope Nef134-10, the Y-to-F mutation (Y135F) at the second position is observed at high frequencies in circulating HIV-1 sequences in Japan – in fact it represents the consensus at this position in Japan - presumably as a result of high HLA-A*24:02 prevalence in the population [4,23]. Our experiments using a Nef134-10-specific CTL clone and a minigene corroborated the earlier observation that the Y135F mutation disrupts antigen processing of the Nef134-10 epitope (Figure 3C) [4,12]. Importantly, while the majority of patients with Y135F responded to the upstream epitope Nef126-10, none of the patients with the wild-type sequence responded to this epitope. Consistent with this observation, results of the peptide binding (Figure 2A) and limiting dilution experiments using antigen-specific CTLs (Figure 2B) were compatible with the previous reports indicating that F, but not Y, could serve as a C-terminal anchor [16,17]. Also consistent with this observation is that the 2nd position of Nef126-10 is Y, a strong N-terminal anchor amino acid for HLA-A*24:02. Taken together, in a process similar to the ongoing exposure of novel antibody epitopes in HIV-1 envelope as a consequence of escape from earlier humoral responses [10], our results demonstrate that an analogous phenomenon also occurs with CTL responses: in this case a novel A*24:02-restricted “epitope switch” from Nef134-10 to Nef126-10, as a result of immune-driven escape at a single Nef codon.

We also showed that Nef residues I133T and Y135F are highly significantly linked *in vivo*. Nef126-10 emerges as a CTL epitope by the introduction of the Y135F mutation. Though I133T has previously been identified as an HLA-A*24:02-associated polymorphism in statistical association studies [4,8,21,22,24,25], its mechanism remained unknown. Our data strongly suggest that I133T is HLA-A*24:02-restricted escape mutation whose mechanism of action is alteration of the *in vivo* CTL repertoire capable of recognizing the HLA-bound epitope. Although the sample size was limited, patients with 133I/135F viruses did not exhibit responses to Nef126-10(8T10F) (Figure 6B). These results, together with studies of a patient whose plasma viral sequences shifted from wild-type to 133T/135F, strongly suggest that immune pressures selected an I-to-T substitution at Nef’s 133rd position. IFN-γ ELISpot assays showed that Nef126-10(8I10F)-specific but not Nef126-10(8T10F)-specific responses correlated inversely with pVL (Figure 5C) and that the former had significantly higher functional avidities (Figure 6C). These findings therefore suggest a TCR-mediated mechanism underlying HLA-A*24:02-mediated escape via I133T. Although higher functional avidity is a hallmark of CTLs with stronger selective pressure [19,20], further studies are needed to confirm that the I133T mutation alleviates immune pressures directed on the Nef126-10 epitope.

Crystal structures of peptide-HLA showed that the side chain of the 133rd residue (P8 residue in the Nef126-10 epitope) protruded from the peptide-binding cleft presumably providing a feature of the Nef126-10 epitope to the TCRs (Figure 6A). The shorter side chain of T compared to I might make the Nef126-10(8T10F) less accessible to TCR than the Nef126-10(8I10F) epitope. Considering the similarity of the structures, the absence of the T cell repertoire against the Nef126-10(8T10F) epitope in the patients with 133I/135F viruses is an enigma. The suggested structural flexibility of the central portion (P5-P7) and P9 of the Nef126-10 epitope may be relevant here.

A key remaining question is why the Y135F mutation is repeatedly selected by A*24:02, given that a consequence of this escape is the introduction of another A*24:02 epitope immediately upstream. We offer the following hypothesis. In studies of HIV-1 infected populations around the globe, the association between HLA-A*24:02 and Nef-135F consistently ranks among the strongest in the HIV proteome [8,21], including in Japan where F (rather than the global subtype B consensus Y) represents the consensus at this position [4,22]. Indeed, a recent international cohort study revealed an odds ratio of >28 and a p-value of 8x10^-118^ for this association [21]. The extraordinary magnitude of this association indicates that Nef-135 is under similarly extraordinary selection pressure by A*24 *in vivo* - presumably due to highly effective CTL responses against the Nef134–10 epitope. The benefits to HIV of evading A*24-mediated recognition of Nef134–10 presumably outweigh its substantial negative consequences to the virus, which in this case include the creation of the adjacent Nef126-10 epitope. That Nef126-10 is targeted by less than 70% of A*24-expressing persons harboring 135F is consistent with escape at this position affording clear viral advantage in at least some cases.

We therefore propose the following model of HLA-A*24:02-mediated CTL targeting of these overlapping Nef epitopes *in vivo*. If an HLA-A*24:02-expressing patient were to be infected with the wild type virus, CTL responses would first arise against Nef134-10, eliciting the Y135F escape mutant. This in turn would reveal the novel Nef126-10 epitope, against which CTL responses would then arise. Similarly, if an HLA-A*24:02 expressing patient were to be infected with a virus harboring Y135F (a likely occurrence given its extremely high prevalence in Japan), we infer that a CTL response against Nef126-10 epitope would be launched immediately following infection.

As such HIV’s evasion of Nef134-10-specific CTL (either via transmission or *in vivo* selection of Y135F) yields a major, albeit temporary advantage to HIV, that is subsequently diminished by the creation of the Nef126-10 epitope that is then targeted in most A*24:02-expressing persons. By extension, the “epitope switching” from Nef134-10 to Nef126-10 may contribute in part to the previous observation of relatively stable pVL over time among HLA-A*24:02 positive patients [26] despite high transmission frequency of 135F [4]. These observations are not inconsistent with the idea that population-level HIV adaptation to HLA could lead to weakening of host antiviral control if “HLA-adapted” forms dominated the population (as is the case with Nef-135F in Japan, which represents the consensus at this position) [27]. If the consensus at Nef codon 135 was the susceptible Y rather than the escaped F in Japan, we hypothesize that control of HIV by A*24 would be even greater in this region, as Nef134–10 and Nef126-10 would be sequentially targeted (rather than just Nef126-10 in the case where 135F is acquired at transmission).

Of note, among the 46 patients in the IMSUT cohort, there were no significant differences in plasma HIV load between patients with or without Nef126-10-specific CD8+ T cell responses (Figure 1, and data not shown). However, this observation should be interpreted in the context that all patients responding to Nef126-10 responses harbored Nef-135F (that confers escape from responses against the downstream Nef134-10 epitope). Moreover, the observation that no reversion of 135F to 135Y was ever observed in Nef126-10 responders (Figure 1C) suggests that Nef126-10-specific CTL responses are not as effective as Nef134-10-specific ones in controlling HIV *in vivo*. Elucidating the mechanisms and *in vivo* relevance of our observations would be crucial for vaccine development.

Among the HLA-associated polymorphisms, amino acid substitutions between K-R, E-D, V-I, I-L, and Y-F are relatively frequent [8,24,25]. The similarities of these amino acid residue pairs with respect to size, charge, hydrophobicity, and other biochemical properties suggest that they are often critical to the structure and function of the viral proteins involved. Although in the present study we characterized an example of escape-induced “epitope switching” in HIV-1, we do not know how often this phenomenon occurs. If an HLA-driven escape mutation has the potential to serve as an anchor of a cryptic epitope, and if an N-terminal or C-terminal anchor residue is present at a proper distance, incorporation of both the wild-type and the mutant peptide in a vaccine may elicit immune response to that cryptic epitope. The vaccine-induced CTL repertoire may be useful following selection of the escape mutant or may prevent its selection altogether. As such, the phenomenon of escape-induced “epitope switch” may be relevant to vaccine design.

## Conclusions

Our data represent the first example of the *de novo* creation of a novel overlapping CTL epitope as a direct result of HLA-driven immune escape in a neighboring epitope. The robust targeting of Nef126-10 following transmission (or *in vivo* selection) of HIV-1 containing Y135F may explain in part the previously reported stable plasma viral loads over time in the Japanese population, despite the high prevalence of both HLA-A*24:02 and Nef-Y135F in circulating HIV-1 sequences.

## Methods

### Patients and samples

The samples and host/viral genotype data analyzed in this study were obtained from three independent sources: (i) the IMSUT cohort at the Institute of Medical Science, the University of Tokyo, Japan, (ii) the baseline (pre-therapy) cross-section of the HOMER cohort in British Columbia, Canada ([8] and unpublished), and (iii) a longitudinal multicenter cohort of acute/early infected individuals [28]. The IMSUT cohort, which consists primarily of Asian patients with chronic infection, was used to determine the viral sequences and immune responses.

#### ***IMSUT cohort***

Forty-six HLA-A*24:02-positive, antiretroviral-naïve, chronically HIV-infected subjects were selected from among patients participating in an ongoing HIV-1-immunopathogenesis study at an HIV outpatient clinic affiliated with the Institute of Medical Science, the University of Tokyo (IMSUT). Study procedures included routine collection of blood samples for virologic and immunologic testing. Peripheral blood mononuclear cells (PBMCs) and plasma samples were separated and preserved in liquid nitrogen or at -80°C, respectively, until use. The study was approved by the internal review board of the Institute of the Medical Science of the University of Tokyo (No. 11–2), and all subjects were adults and provided written informed consent.

#### ***HOMER cohort***

A total of 1038 patients from the HAART Observational Medical Evaluation and Research (HOMER) cohort, an open cohort of initially antiretroviral-naïve chronically HIV-infected individuals in British Columbia, Canada of predominantly Caucasian ethnicity, were analyzed in the present study. Plasma HIV-1 RNA sequencing and HLA class I sequence-based typing were performed as previously described [8]. We applied phylogenetically-corrected methods [25] to determine the strength of association between amino acid variants at Nef codons 133 and 135 in this dataset.

#### ***Longitudinal acute/early infection cohort***

Kaplan-Meier analysis was used to investigate the time course of selection of specific immune escape mutations at Nef codons 133 and 135 among 16 HLA-A*24 expressing individuals from a longitudinal, multicenter, acute/early HIV-1 infection cohort [28]. “Time to escape” was defined as the number of days elapsed between estimated infection date and first detection of the escape variant (as a full or partial amino acid change).

### Plasma viral RNA sequences

Viral RNA was extracted from 140 μl of plasma using the QIAamp viral RNA Mini kit (QIAGEN). Using 4 μl of RNA as starting material, reverse transcription and first PCR were carried out according to the manufacturer’s protocol with SuperScript III One-Step RT-PCR System with Platinum Taq High Fidelity (Invitrogen). Two μl of the first PCR product was subjected to nested PCR, performed using Ex-Taq HS (Takara) with 35 cycles of 30s at 94°C, 30s at 55°C, 60s at 72°C and a final extension for 7 min at 72°C. The primer sets were as follows (Nucleotide positions are those of the published HIV-1 SF2 strain (GenBank accession number: K02007). For the first PCR, primers Nef-1F (5’-GTAGCTGAGGGGACAGATAGGGTTAT-3’) (nt 8,688 to 8,731) and Nef-1R (5’-GCACTCAAGGCAAGCTTTATTGAGGC-3’) (nt 9,632 to 9,607) were used; and for the nested PCR, primers Nef-2F (5’-CGTCTAGAACATACCTAGAAGAATAAGACAGG-3’) (nt 8,746 to 8,777) and Nef-2R (5’-CGGAATCCGTCCCCGCGGAAAGTCCCTTGTA-3’) (nt 9,477 to 9,444) were used. The PCR products were purified with a PCR purification kit (QIAGEN) before sequencing. DNA sequencing was performed using an ABI Prism dye terminator cycle sequencing-ready reaction kit (Applied Biosystems) on a Perkin-Elmer ABI-377 sequencer.

### Expression vectors

To construct the HLA-A*24:02 expression vector, pcDNA3.1-A24-DsRedm, the HLA-A*24:02 sequence was amplified by PCR using cloned HLA-A*24:02 cDNA as a template [29,30] and digested with BamHI and NcoI. Primers (5’-TAATACGACTCACTATAGGG-3’) and (5’-CCATGGATCCGCCCCCTCCCACTTTACAAGCTGTGAGAGACAC-3’) were used for the amplification. The pDsRed-Monomer (Clontech) sequence was digested by NcoI and NotI, and was ligated to the 3’ end of HLA-A*24:02 fragment to obtain the HLA-A24-DsRedm fragment. Then HLA-A24-DsRedm fragment was inserted into the multiple cloning site of pcDNA3.1/Hygro(+) vector (Invitrogen).

Mini-Nef and mini-Gag gene expression vectors containing two reporter genes, renilla luceferase (Rluc) and EGFP, together with hygromycin selection were constructed in a pTracer-CMV2 vector (Invitrogen) as follows. Wild-type and mutant mini-Nef genes (from amino acid position 123 to 153) were amplified by PCR using the plasmids containing HIV-1 SF-2 Nef gene with wild-type or mutant sequence as a template [4,31,32]. Primer sequences were (5’-GGTACCGCCGCCATGGATTGGCAGAATTACACA-3’) and (5’-GGATCCGCCCCCTCCTACCTTCTCTGGCTC-3’). A mini-Gag gene, extending from amino acid position 18 to 46 in p17, which includes the HLA-A*24:02-restricted CTL epitope Gag28-9 (KYRLKHIVW), was amplified using a plasmid containing the 5’ half of the HIV-1 SF2 strain [31,32]. The primers used were 5’-GGTACCGCCGCCATGAAAATTCGGTTAAGG-3’ and 5’-GGATCCGCCCCCTCCGACTGCGAATCGTTC-3’. The Rluc and hygromycin genes were amplified from pGL4.77 hRlucP/Hygro (Promega) using primers 5’-GGATCCATGGCTTCCAAGGTGTAC-3’ and 5’-TCTAGAGTCGCGGCCTTAGACGTT-3’.

KpnI/BamHI fragments of mini-Nef or mini-Gag, BamHI/XbaI fragment of Rluc and hygromycin genes amplified from pGL4.77 hRlucP/Hygro were ligated to the KpnI/XbaI fragment of pTracer-CMV2 vector (Invitrogen) to create a mini-Nef or mini-Gag expression vector, pmNef(wt)-hRluc-EGFP and pmGag(wt)-hRluc-EGFP, respectively. In the final step the GFPz sequence was replaced by EGFP sequence (Clontech).

### Peptides

Synthetic peptides were purchased from Sigma-Genosys. The peptides used in the screening of immune response by ELISpot had a purity of 70% or more. All other peptides were more than 95% pure as determined by high-performance liquid chromatography and mass spectroscopy.

### Cells and media

T2-A24, a kind gift from K. Kuzushima, was cultured in RPMI 1640 (Sigma) supplemented with 100 U of penicillin/ml, 100 U of streptomycin/ml, 10% heat-inactivated fetal calf serum (FCS) (Sigma), and 0.8 mg of G418 (Invitrogen)/ml [33]. We established Nef126-10 and Nef134-10-specific CTL clones, I30-1 and H27-9, as previously described [18]. CTL clones were cultured with RPMI 1640 supplemented with 50 U of interleukin-2/ml, 100U of penicillin/ml, 100U of streptomycin/ml, and 10% heat-inactivated FCS (R10/50), but the clones were cultured in the absence of interleukin-2 (R10) for two days before antigen presentation assays. pcDNA3.1-A24-DsRedm was introduced into 293FT cell line (Invitrogen) and the cells were treated by hygromycin for 2 weeks. After cloning by limiting dilution we obtained 293FT-A24DRm-CY0, and confirmed HLA-A*24:02 expression with FACS analysis by using anti-HLA-A9 serotype antibody (One Lambda, data not shown).

### IFN-γ ELISpot assay

The gamma interferon enzyme-linked immunospot (IFN-γ ELISpot) assay was performed using patients’ PBMCs as previously described [4] with some modifications. In brief, 96-well plates (Millipore) were coated with anti-gamma-interferon (IFN-γ) MAb 1-D1k (Mabtech) overnight at 4°C. Peptides were added directly to the wells at a final concentration of 10^-5^ M. 5 ~ 10 × 10^4^ cells were added to each well with a final volume of 100 μl of R10. For negative controls, PBMCs were incubated with R10 alone without peptides. After incubation at 37°C under 5% CO_2_ overnight (16 to 18 h), the plates were washed six times with phosphate-buffered saline containing 0.01% tween-20 (PBST). Biotinylated anti-IFN-γ MAb 7-B6-1 (Mabtech) was added, and was incubated for 2 hours at 37°C under 5% CO_2_. After washing with PBST, streptavidin-alkaline phosphatase conjugate (Mabtech) was added and the plates were kept at room temperature for 45 min. After washing with PBST, IFN-γ-producing cells were detected as dark spots after 10- to 20-min color reaction with 5-bromo-4-chloro-3-indolylphosphate and nitroblue tetrazolium by using AP Conjugate Substrate Kit (Bio-Rad). Spots were counted by KS ELISPOT compact (Carl Zeiss) and expressed as spot-forming units (SFU) per 10^6^ PBMCs after subtracting the SFU of the negative control. Values with >50 SFU, >3 × mean SFU of negative control and > mean SFU of negative control + 3 SD per 10^6^ input cells were considered as a positive response.

Since more cells were required for the immune response screening (Figure 1B) and functional avidity assays (Figures 4B and 6C), PBMCs were stimulated with anti-human CD3 antibody and the T cells were expanded for 2 to 3 weeks in R10/50 (BD Pharmingen). Culture media was changed to R10 two days prior to the assay date. For *ex vivo* IFN-γ ELISpot assay (Figures 5B and 6B), PBMCs were cultured for 6 hours in R10 media.

### Peptide-HLA binding assay (Figure 2A)

Peptide binding to HLA-A*24:02 was assessed by using a T2-A24 stabilization assay as previously described [4,33]. Briefly, after incubation for 16 hours at 26°C under 5% CO_2_, 2 × 10^5^ T2-A24 cells were incubated with 10^-4^ to 10^-9^ M peptides for 1 h at 4°C. After keeping at 37°C under 5% CO_2_ for 3 hours, the cells were stained with biotinylated anti-human HLA-A9 monoclonal antibody (One Lambda), and streptavidin-APC conjugates (BD Pharmingen). The mean fluorescence intensity (MFI) was measured by FACSCalibur (Becton Dickinson). In each experiment, MFI of samples was normalized by the MFI of 10^-4^ M control peptide, Nef134-8(RYPLTFGW). Three independent experiments were performed.

### CTL clones and Epitope recognition (Figure 2B)

Nef134-10- and Nef126-10-specific clones, H27-9 and I30-1, were established by Nef134-10(wt) and Nef126-10(wt) peptide stimulation respectively, and limiting dilution of PBMCs from HIV-1-infected patients harboring 133 T/135 F. For *in-vitro* peptide stimulation, 5 × 10^5^ PBMCs were pulsed with 10 μM of each peptide for 1 h. The cells were washed twice with R10, then cultured with 1 × 10^6^ autologous PBMCs and 4 × 10^6^ irradiated (3300 rad) allogeneic PBMCs in R10. After 4 days, IL-2 was added to 50 U/ml and the cells were cultured for 2–3 weeks in R10/50. Peptide-specific CD8^+^ T cells were enriched by MACS separation (Miltenyi) using tetramers. The sorted cells were cloned by limiting dilution to 3 or 10 cells/well in 96-well round-bottom tissue culture plates, and cultured with 10^5^ irradiated allogeneic PBMCs in R10/50 containing 5 μg/ml PHA-L.

CTL-recognition of the epitopes was assessed by serially diluted peptides. On day 0, 293FT-A24DRm-CY0 cells were seeded onto 96-well Flat-bottom transparent plate (BD Falcon) so that the cultures become confluent on day 2. On day 2, each peptide was pulsed with concentrations from 3^2^ to 3^-6^ μM to the wells and incubated at 37°C under 5% CO_2_ for 1 h. Then CTLs (5,000 ~ 10,000 cells) were added and co-cultured at 37°C under 5% CO_2_ for 18 to 24 h. After the incubation, supernatants were harvested, and IFN-γ concentrations were quantified by Human IFN-γ ELISA Set (BD Bioscience). In each experiment, the IFN-γ value of samples was normalized to that of the highest wild type peptide concentration (9 μM). For example, each IFN-γ value of I30-1 CTL clone was divided by the value of the well pulsed with 9 μM Nef126-10(wt) peptide. Each assay was performed in duplicate and three independent experiments were conducted.

### Antigen presentation assay (Figure 3)

Antigen presentation was assessed by measuring epitope-specific CTL responses to endogenously expressed antigen. First, intracellular expression of each antigen was extrapolated from the activity of reporter protein, Rluc. 293FT-A24DRm-CY0 cells were seeded in 96-well plate (Nunc) on day 0. On day 1, antigen expression plasmids, pmNef(wt)-hRluc-EGFP, pmNef(135F)-hRluc-EGFP, pmNef(133T135F)-hRluc-EGFP, or control vectors (pmGag(wt)-hRluc-EGFP) were transfected into the cells in each well using FuGENE HD (Promega). The cultures were incubated at 37°C under 5% CO_2_ for 18 to 24 h. On day 2, transfection efficiency was inspected roughly under a fluorescence microscope (KEYENCE BZ-9000), then Rluc activity in each transfected well was measured by using Dual-Glo Luciferase Assay System (Promega) and luminometer (Promega GloMax 96 Microplate Luminometer). The assay was performed in triplicate.

Second, CTL responses against endogenously expressed and processed epitopes were evaluated. 293FT-A24DRm-CY0 cells were seeded onto a 96-well Flat-bottom transparent plate (BD Falcon) on day 0. On day 1, expression plasmid was transfected to each well using FuGENE HD (Promega). CTLs (5,000 ~ 10,000 cells) were added to the transfected wells on day 2. After incubation for 18 to 24 h, the supernatant of each well was harvested, and IFN-γ secretion was quantified by Human IFN-γ ELISA Set (BD Bioscience). IFN-γ ELISA was performed in duplicate.

Experiments were performed in duplicate on three independent occasions. To normalize the values in each experiment, mean IFN-γ values of each sample were normalized by the mean value of reference wells in duplicate. In the reference wells, 9 μM of the wild type peptide was pulsed to the antigen presenting cells and then co-cultured with CTLs.

### Protein expression in E. coli, refolding and purification

HLA-A*24:02 and β2m were expressed in *E. coli* and refolded from inclusion bodies as previously described with some modifications [34]. HLA-A*24:02 (18 mg), β2m (6 mg) and peptide (4 mg) were mixed in 400 ml of refold buffer containing 100 mM Tris, pH 8.0, 400 mM L-arginine-HCl, 2 mM EDTA, 5 mM GSH, 0.5 mM GSSG, 0.2 mM PMSF. The refolded protein was purified by Superdex 75 column, followed by Mono Q column, and subsequently concentrated to 10 mg/ml in 20 mM Tris, pH 8.0, 50 mM NaCl for crystallization.

### Crystallization, data collection and structure determination

The crystallization was done by the sitting drop vapor diffusion method at 20°C. Crystals of the A24/N126-10(8T10F) complex were obtained in 20% (w/v) PEG 3350, 200 mM sodium phosphate dibasic, and those of A24/N126-10(8I10F) were obtained in 20% (w/v) PEG 3350, 200 mM sodium nitrate. For cryoprotection, crystals were soaked briefly in reservoir solutions containing 20% ethylene glycol, and then frozen in liquid nitrogen before data collection. Data were collected at the beamline BL41XU in SPring 8 (Hyogo, Japan), and processed with HKL2000 [35] and the CCP4 program suite [36].

The structure were determined by molecular replacement using Molrep [37]. The search model was the coordinate file of PDB (Protein Data Bank) code 3I6L with omitted peptide for A24/N126-10(8T10F). Model building and refinement were carried out using Coot [38] and REFMAC5.6 implemented in CCP4, respectively. The structure of A24/N126-10(8I10F) was determined with the refined A24/N126-10(8T10F) as a search model and refined as described above. The stereochemistry of the refined models was assessed with RAMPAGE [39]. All molecular graphic representations were created with the program PyMOL (DeLano Scientific; http://www.pymol.org). Data collection and refinement statistics are shown in Additional file 2: Table S1.

### Functional avidity assay

Basically, the assay was performed using the same procedure with IFN-γ ELISpot assay as described above. PBMCs were cultured for 2 to 3 weeks in R10/50 after anti-human CD3 antibody (BD Pharmingen) stimulation, and culture media were changed from R10/50 to R10 two days before use. PBMCs were incubated with peptides at concentrations from 10^-5^ to 10^-12^ M, and SFU was calculated. The functional avidity to peptide dilutions was determined as a 50% of sigmoidal dose (SD_50_) SFU.

### Statistical analysis

All data visualization and statistical analyses were performed using GraphPad Prism (GraphPad Software, La Jolla, CA). Student’s t-test and Mann–Whitney U-test were used to compare the antigen presentation and functional avidity between two groups, respectively. Spearman rank correlation was used to calculate the correlation between peptide-specific response and pVL. Dose at 50% response in sigmoidal dose–response curves (SD_50_) was calculated by drawing sigmoidal dose–response curves. Time to mutational escape, defined as the time elapsed between estimated date of HIV-1 infection and the first appearance of a full or partial amino acid change consistent with the specific escape mutation of interest, was calculated using Kaplan-Meier (survival) methods.

## Competing interests

AI. has received grant support from Toyama Chemical Co. Ltd., astellas, ViiV Healthcare KK., MSD KK., Baxter through the University of Tokyo. AI. has received speaker’s honoraria/payment for manuscript from Eiken Chemical Co. Ltd., astellas, Toyama Chemical Co. Ltd, Torii Pharmaceutical Co. Ltd., MSD KK., and Taisho Toyama Pharmaceutical Co. Ltd. The authors have no additional financial interests.

## Authors’ contributions

CH conceived of the study, carried out the molecular genetic studies and immunoassays, and drafted the manuscript. AKT established CTL clones and construction of expression vector. AS, YS, AY and SF carried out protein synthesis and solved crystal structures. DZ contributed to the sequence analysis. HN, EA, TKi, MK and TKo provided the clinical materials and data of the IMSUT cohort. GFG participated to the study design. EM and ZLB investigated the relationship between HLA-A*24 expression and sequence variants at Nef codons 135 and 133 in 1018 participants in British Columbia HOMER cohort and longitudinal multicenter acute/early HIV-1 infection cohort with HIV-1 Nef and HLA-A data available. ZLB helped to draft the manuscript. AI participated in the study design, coordination and helped to draft the manuscript. All authors read and approved the final manuscript.

## Supplementary Material

Additional file 1: Figure S1 Overview of structures of the HLA-A*2402 in complex with the Nef126-10 peptides. Structures of **(A)** the A24/N126-10(8I10F) and **(B)** the A24/N126-10(8T10F).The electron density of **(C)** the Nef126-10(8I10F) and **(D)** the Nef126-10(8T10F) are shown with *F*_o_-*F*_c_ omit maps contoured at 2.0 σ (cyan mesh). (**C** and **D**) The peptide structures are shown in a side view (top panels) and top view (bottom panels). The Nef126-10 (8I10F) and the Nef126-10 (8T10F) are shown as a purple and a green stick model, respectively. HLA-A24 and β2m are represented as gray and black cartoon model, respectively. Click here for file

Additional file 2: Table S1 Data collection and refinement statistics. Click here for file

## Accession numbers

The coordinates and structure factors of the HLA-A24/Nef126-10(8I10F) and HLA-A24/Nef126-10(8T10F) have been deposited [Protein Data Bank: 3WL9 and 3WLB, respectively].
