# Supplementary figures and images for: Switching and emergence of CTL epitopes in HIV-1 infection
Source: Retrovirology. 2014 May 21;11:38. doi: 10.1186/1742-4690-11-38 (PMC4036671; doi:10.1186/1742-4690-11-38)

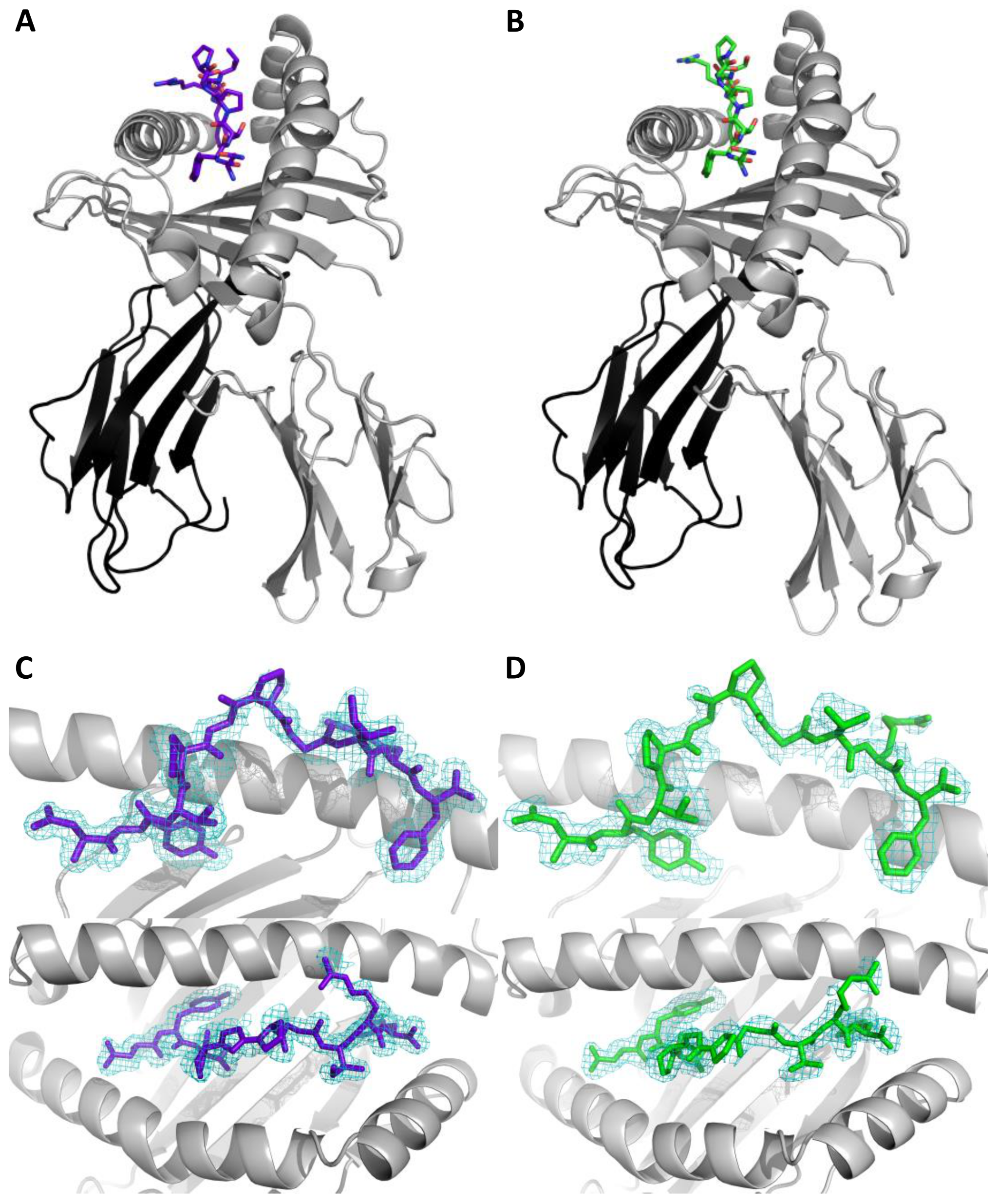

Supplement: Additional file 1: Figure S1 — Overview of structures of the HLA-A*2402 in complex with the Nef126-10 peptides. Structures of (A) the A24/N126-10(8I10F) and (B) the A24/N126-10(8T10F).The electron density of (C) the Nef126-10(8I10F) and (D) the Nef126-10(8T10F) are shown with Fo-Fc omit maps contoured at 2.0 σ (cyan mesh). (C and D) The peptide structures are shown in a side view (top panels) and top view (bottom panels). The Nef126-10 (8I10F) and the Nef126-10 (8T10F) are shown as a purple and a green stick model, respectively. HLA-A24 and β2m are represented as gray and black cartoon model, respectively. [file 1742-4690-11-38-S1.tiff]
