# Supplementary material for: Switching and emergence of CTL epitopes in HIV-1 infection
Source: Retrovirology. 2014 May 21;11:38. doi: 10.1186/1742-4690-11-38 (PMC4036671; doi:10.1186/1742-4690-11-38)
Supplement: Additional file 2: Table S1 — Data collection and refinement statistics. [file 1742-4690-11-38-S2.pdf]

	A24/N126 (8I10F)	A24/N126 (8T10F)
**Data collection**		
Space group	$P2_12_12_1$	$P2_12_12_1$
Cell dimensions		
a, b, c (Å)	66.58, 78.41, 87.28	67.08, 77.73, 87.10
α, β, γ (°)	90, 90, 90	90, 90, 90
Resolution (Å)		
R_{sym}	0.084 (0.425)	0.086 (0.318)
I/σ	23.4 (2.4)	24.6 (3.8)
Completeness (%)	99.1 (97.5)	99.0 (98.2)
Redundancy	5.4	5.2
**Refinement**		
Resolution (Å)	50-1.66	50-2.00
No. reflections	51,407	29,606
R_{work}/R_{free} (%)	17.5/22.6	18.0/24.7
No. atoms		
Protein	3,139	3,138
Water	519	282
B-factors (Å^2)		
Protein	23.03	30.25
Water	33.06	37.43
R.m.s. deviations		
Bond lengths (Å)	0.007	0.007
Bond angles (°)	1.064	1.186
Ramachandran plot		
Favoured (%)	98.7	98.1
Allowed (%)	1.3	1.9

Values in parentheses are for highest-resolution shell.
